# Supplementary material for: Potential Oral Microbial Markers for Differential Diagnosis of Crohn’s Disease and Ulcerative Colitis Using Machine Learning Models
Source: Microorganisms. 2023 Jun 26;11(7):1665. doi: 10.3390/microorganisms11071665 (PMC10385744; doi:10.3390/microorganisms11071665)
Supplement: Supplementary file 1 [file microorganisms-11-01665-s001.zip › microorganisms-2451477-supplementary.pdf]

Table S1. Top10 genera selected with high frequency in the IBD vs HC sPLS-DA models.

Genera contributing to HC	Frequency	Genus contributing to IBD	Frequency
Ferritrophicum	100	*Puniceicoccaceae_A714019*	75
Candidatus Curculioniphilus	97	*Elizabethkingia*	66
Candidatus Puchtella	96	*Sporocytophaga*	49
Pricia	94	*Zhihengliuella*	44
Paludibacteraceae_F0058	93	*Catellatospora*	38
Lachnoanaerobaculum	93	*Zunongwangia*	27
Lachnobacterium	93	*Rhodocytophaga*	26
Marininema	93	*Zeaxanthinibacter*	25
Sandaracinaceae_uncultured	93	*Tunicatimonas*	20
Candidatus Moranella	89	*Marinilutecoccus*	19

Table S2. Top10 genera selected with high frequency in the CD vs UC sPLS-DA models.

Genera contributing to CD	Frequency	Genus contributing to UC	Frequency
Candidatus Anadelfobacter	95	*[Eubacterium] hallii group*	95
Candidatus Fokinia	95	*Annamia HOs24*	95
Chitinimonas	95	*Lachnospiraceae AC2044 group*	95
Thiomicrospira	95	*Marvinbryantia*	95
Bythopirellula	94	*Vibrionaceae_uncultured*	95
Collimonas	94	*Actinomadura*	94
Desulfuromonas	94	*Desulfurispira*	94
Actimicrobium	93	*Thermobrachium*	94
Limibacillus	92	*Clostridium sensu stricto 1*	88
Candidatus Defluviella	88	*Lachnospiraceae NC2004 group*	87
